# Predictors of intra-hospital mortality in patients with diabetic foot ulcers in Nigeria: data from the MEDFUN study

**DOI:** 10.1186/s12902-020-00614-4

**Published:** 2020-08-28

**Authors:** Olufunmilayo O. Adeleye, Ejiofor T. Ugwu, Ibrahim D. Gezawa, Innocent Okpe, Ignatius Ezeani, Marcelina Enamino

**Affiliations:** 1 Department of Medicine, Lagos State University Teaching Hospital Ikeja, Lagos, Nigeria; 2 Department of Medicine, Enugu State University of Science and Technology Enugu, Enugu, Nigeria; 3 Department of Medicine, Bayero University Kano, Kano, Nigeria; 4 Department of Medicine, Ahmadu Bello University Zaria, Zaria, Nigeria; 5 Department of Medicine, Federal Medical Center Umuahia, Umuahia, Abia Nigeria; 6 Department of Medicine, Federal Medical Center Keffi, Keffi, Nasarawa Nigeria

**Keywords:** Diabetic foot ulcers, Predictors, Intrahospital mortality, Wagner grading, Sepsis, Renal impairment

## Abstract

**Background:**

Diabetic foot ulcers (DFU) are associated with high morbidity and mortality globally. Mortality in patients hospitalized for DFU in Nigeria is unacceptably high. This study was undertaken to determine factors that predict mortality in patients hospitalized for DFU in Nigeria.

**Methods:**

The current study was part of Multi-centre Evaluation of Diabetic Foot Ulcer in Nigeria (MEDFUN), an observational study conducted in six tertiary healthcare institutions across the 6 geopolitical zones of Nigeria. Consecutive type 1 or 2 diabetic patients hospitalized for DFU who consented to participate were recruited and subjected to relevant clinical, biochemical, and radiological assessments and multidisciplinary care until discharge or death. Data for type 1 diabetes mellitus (DM) patients were expunged from current mortality analysis due to their small number.

**Results:**

Three hundred and twenty-three type 2 DM subjects with mean age and mean duration of DM of 57.2 ± 11.4 years and 8.7 ± 5.8 years respectively participated in this study. The median duration of ulcers was 39 days with a range of 28 to 54 days and the majority (79.9%) presented with advanced ulcers of at least Wagner grade 3. Mortality of 21.4% was recorded in the study, with the highest mortality observed among subjects with Wagner grade 5.

Variables significantly associated with mortality with their respective *p* values were DM duration more than 120 months (*p* 0.005), ulcer duration > 1 month (*p* 0.020), ulcer severity of Wagner grade 3 and above (*p* 0.001), peripheral arterial disease (*p* 0.005), proteinuria (*p* < 0.001), positive blood cultures (*p* < 0.001), low HDL (*p* < 0.001), shock at presentation (*p* < 0.001), cardiac failure (*p* 0.027), and renal impairment (*p* < 0.001). On Multivariate regression analysis, presence of bacteraemia (OR 5.053; 95% CI 2.572–9.428) and renal impairment (OR 2.838; 95% CI 1.349–5.971) were significantly predictive of mortality independent of other variables.

**Conclusions:**

This study showed high intra-hospital mortality among patients with DFU, with the majority of deaths occurring among those with advanced ulcers, bacteraemia, cardiac failure, and renal impairment. Prompt attention to these factors might help improve survival from DFU in Nigeria.

## Background

Diabetic foot ulcer (DFU) is one of the most debilitating chronic complications of diabetes mellitus (DM) and its prevalence is increasing exponentially across the globe. The number of people with diabetes worldwide is currently estimated to be 425 million with this number projected to increase to 629 million by year 2045 [1].The implication of this increase is a corresponding rise in micro and macrovascular complications of the disease. Every thirty seconds, a lower limb or part of it is lost to amputation as a consequence of diabetes globally [2]. Diabetic foot ulcer is characterized by poor short and long term survival especially when associated with other microangiopathic comorbidities [2–6]. Mortality in hospitalized diabetic patients with DFU is quite considerable compared to those without. One of the commonest causes of death among hospitalized diabetic patients is DFU. Mortality due to DFU was reported to be 14% in Africa and 40.5% in a cohort of Nigerians [7–9]. Although there is the paucity of data regarding the factors associated with intra-hospital mortality in Nigerian patients with DFU, reports from other parts of the world indicate that lower extremity amputation (LEA) conferred a higher risk of mortality [6]. Malnutrition, confirmed using a geriatric nutritional assessment risk index was found to be associated with higher mortality among patients admitted for DFU in concert with older age and reduced glomerular filtration rate [10, 11].The Site of osteomyelitis (hind versus mid/forefoot) was a determinant of higher mortality in a cohort of DFU patients in Arezzo, Italy [12]. Other factors that promote the higher risk of mortality in patients hospitalized for DFU are a raised white cell and neutrophil count, ulcer severity at presentation and anemia [7, 13, 14]. Given the currently unacceptably high mortality in patients admitted for DFU in Nigeria [7], it is important to identify contributory factors which will predict outcome in this group of patients. Identification of these factors will enable optimized management strategy and stem this tide of high DFU related mortality. Prognostic predictors will identify patients in need of more intensive monitoring and treatment. This study is to document those factors associated with mortality in patients admitted for DFU in Nigeria.

## Methods

This analysis is from The Multi-centre Evaluation of Diabetic Foot Ulcer in Nigeria (MEDFUN), an observational study, which was conducted across Nigeria, from March 2016 to April 2017. The centres included Lagos State University Teaching Hospital (Southwest Nigeria), Enugu State University Teaching Hospital (Southeast Nigeria), Aminu Kano Teaching Hospital (Northwest Nigeria), Ahmadu Bello University Teaching Hospital Zaria (Northwest Nigeria), Federal Medical Centre Keffi (North Central Nigeria) and Federal Medical Centre Umuahia (Southeast Nigeria). Consecutive type 1 and type 2 diabetic patients hospitalized for DFU during the study period, and who gave verbal consent were recruited by convenience sampling method. Approval of the study protocol was obtained from the local Research and Ethics committee of each of the hospitals.

A detailed protocol of the MEDFUN study has been published elsewhere [15]. Socio-demographic information and relevant clinical history of eligible and consenting subjects were obtained and documented in case report forms designed for the study [15].

The evidence of wound infection was defined as stated by the International Working Group on Diabetic Foot (IWGDF) guideline mainly; the presence of purulent exudates or any two or more of the following: foul smell, local warmth, peri-wound edema, peri-wound redness, pain or tenderness on palpation and fever [16]. Ulcer severity was classified according to Wagner grading of foot ulcers [17]. Peripheral neuropathy was present if there is the loss of pressure sensation in the feet to Semmes-Weinstein 10 g monofilament test or reduced vibration sense utilizing 128 Hz tuning fork. The presence of peripheral artery disease (PAD) and its severity was detected based on - the inability to palpate the dorsalis pedis and/or posterior tibial artery pulsations, or arterial narrowing above 50 % on Doppler ultrasonography of the lower limbs, and ankle-brachial index classified as mild (0.81–1), moderate (0.5–0.8), and severe(< 0. 5) [18] respectively.

The subjects were also evaluated for co-morbidities and presence of DFU related complications including shock (defined as evidence of circulatory failure or blood pressure less than 90/60 mmHg), hypoglycaemia (random capillary blood glucose < 70 mg/dl), anaemia (haemoglobin concentration < 12 g/dl for women and less than 13 g/dl for men), hyperglycaemic emergency (random capillary blood glucose > 450 mg/dl with evidence of fluid depletion and/or metabolic acidosis), stroke, hypertension, cardiac failure, and renal impairment. The latter was diagnosed if serum creatinine was above 1.5 mg/dl or 132 μmol/liter.

Laboratory investigations conducted for each participant include glycated haemoglobin (HbA1c), urine protein, serum creatinine, complete blood count, blood culture, ulcer specimen culture, plain radiograph of the foot, and lipid parameters (total cholesterol (TC), high-density lipoprotein cholesterol (HDL-C), low-density lipoprotein cholesterol (LDL-C) and triglycerides.

Each participant was managed according to the standard protocol of the multi-disciplinary diabetic foot care team in each of the study centres. These protocols which were essentially similar across the different centres include fluid and electrolyte balance, optimum glycaemic control with individualized insulin regimen, wound debridement and dressings as needed, control of wound infection and/or bacteraemia, and limited amputation. Patients were followed up till discharge or death and the outcome of hospitalization was documented. Outcome variables of interest were the duration of hospitalization, wound healing, amputation, and death. The current article was developed from a sub-analysis of mortality data from the MEDFUN study.

### Statistical analysis

Data were analysed with the Statistical Package for Social Sciences (IBM version 23.0; SPSS Inc., Chicago, IL, USA). Numbers and percentages or means and standard deviations were determined for nominal and continuous variables respectively. After excluding subjects who discharged from the hospital against medical advice, univariate logistic regression was conducted for variables of interest to evaluate individual association with mortality by calculating the unadjusted odds ratios (ORs) and 95% confidence intervals (CI). The variables shown to significantly predict mortality at this univariate level of analysis were then simultaneously subjected to a stepwise backward multivariate regression, relying on the Hosmer-Lemeshow goodness of fit test for model reliability. Statistical significance was assumed at *P* < 0.05.

## Results

A total of 336 patients consisting of 13 type 1 DM and 323 type 2DM subjects participated in the study. However, due to this small number (3.9%), type 1 DM subjects were excluded from this analysis. Therefore, data for 323 subjects (54.2% males) with type 2 diabetes are hereby presented. Their mean age was 57.2 ± 11.4 years and mean duration of DM was 8.7 ± 7.8 years. The majority of the subjects (71.5%) did not access routine diabetes care at the study centres and glycaemic control was generally poor with mean HbA1c of 9.6 ± 2.0%. The median (IQR) duration of ulcer before presentation was 39 (28–54) days and 77.1% of ulcers were already infected. Ulcers were adjudged advanced (Wagner grade ≥ 3) in 79.9% of the subjects. Other sociodemographic and clinical characteristics of the study subjects are shown in Table 1.

**Table 1:** Socio-demographic and clinical characteristics of the subjects. Data are presented in n (%) or mean ± SD. *Data are in median (Interquartile range)

Variable	Overall
Age (years)	57.2 ± 11.4
Gender (male)	175 (54.2%)
Diabetes duration (years)	8.7 ± 5.8
Glycated haemoglobin (%) (*n* = 284)	9.6 ± 2.0
Receiving diabetes care at the study centres prior to admission	92 (28.5%)
Ever received foot care education since diagnosis of diabetes	81 (25.1%)
Cigarette smoking (current smokers)	14 (4.3)
Duration of ulcer before admission (days)	39 (28–54)
Previous history of ulcer	91 (28.2%)
Presence of wound infection	249 (77.1%)
Ulcer grade (Wagner)	
Grade 1	12 (3.7%)
Grade 2	53 (16.4%)
Grade 3	82 (25.4%)
Grade 4	122 (37.8%)
Grade 5	54 (16.7%)
Co-morbid complications	
Hypertension	191 (59.1%)
Shock	40 (12.4%)
Anaemia	176 (54.5%)
Hyperglycaemic emergency	118 (36.5%)
Hypoglycaemia	40 (12.4%)
Cardiac failure	23 (7.1%)
Renal impairment	65 (20.1%)
Stroke	32 (9.9%)

**Fig. 1:**
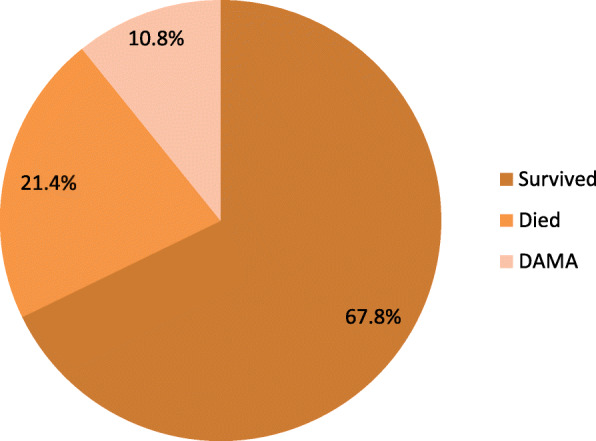
Outcome of diabetic foot ulcer admissions in Nigeria. DAMA = Discharged against medical advice

Figure 1 shows the admission outcomes in the study population. Sixty-nine subjects (21.4%) died during hospitalization while the rest of the population were either discharged after achieving satisfactory wound healing and clinical recovery (67.8%) or discharged against medical advice (10.8%). Subjects who discharged against medical advice shared similar characteristics with those who stayed throughout the admission (data not shown) and the commonest reason for opting out of care was the refusal of amputation (48.6%) followed by financial constraint (42.9%).

**Fig. 2:**
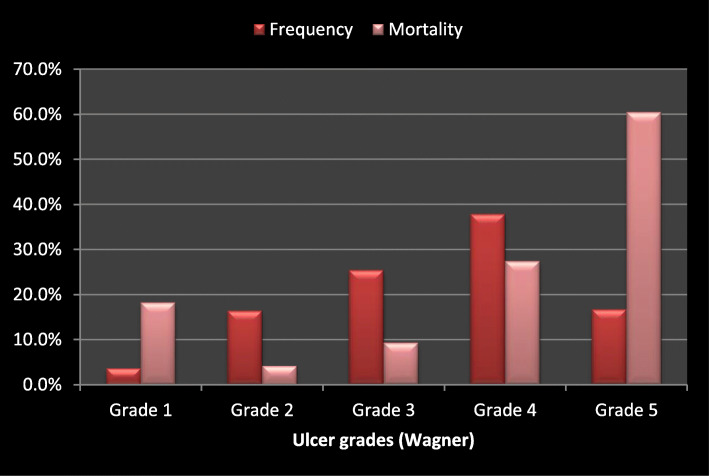
Mortality and grade of ulcer

Mortality increased with ulcer grade, with the highest mortality recorded among subjects with Wagner grade 5 ulcers (Fig. 2). Demographic variables that were significantly related to mortality are presented in Table 2. Mortality was significantly higher among the elderly population aged 65 and above (OR 3.6, *P* 0.045), those with ulcer duration longer than 30 days (OR 2.2, *P* 0.020), and those with Wagner grade 3 ulcer and above (OR 7.6, *P* 0.001). Others include longer duration of DM (*P* 0.005), peripheral artery disease (*P* 0.005), and foot gangrene (*P* < 0.001).

**Table 2:** Relationship between demographic and clinical variables and mortality. Data are in n (%)

	Outcome		*P* value	OR	95% C. I for OR
	Died n (%)	Survived n (%)			
***Age group (years)***					
Elderly (≥ 65)	23 (29.5)	55 (70.5)	**0.045**	3.624	1.997–13.171
Middle-aged (45–64)	43 (23.8)	138 (76.2)	0.117	2.700	0.779–9.361
Young (< 45)	3 (10.3)	26 (89.7)			
***Gender***					
Male	40 (25.6)	116 (74.4)	0.467	1.225	0.709–2.116
Female	29 (22.0)	103 (78.0)			
***Cigarette smoking***					
Ex	8 (23.5)	26 (76.5)	0.873	0.933	0.401–2.172
Current	1 (8.3)	11 (91.7)	0.222	0.276	0.035–2.181
Never	60 (24.8)	182 (75.2)			
***DM duration (months)***					
≤ 120	42 (19.7)	171 (80.3)	**0.005**	0.437	0.245–0.780
> 120	27 (36.0)	48 (64.0)			
***Ulcer > 1 month***					
Yes	56 (27.9)	145 (72.1)	**0.020**	2.198	1.130–4.276
No	13 (14.9)	74 (85.1)			
**Ulcer grade (Wagner)**					
≥ grade 3	66 (28.8)	163 (71.2)	**0.001**	7.558	2.285–24.999
< grade 3	3 (5.1)	56 (94.9)			
***Neuropathy***					
Yes	52 (23.4)	170 (76.6)	0.697	0.882	0.468–1.661
No	17 (25.8)	49 (74.2)			
***Peripheral artery disease***					
Yes	46 (30.9)	103 (69.1)	**0.005**	2.252	1.278–3.969
No	23 (16.5)	116 (83.5)			
***Foot Gangrene***					
Yes	59 (38.3)	95 (61.7)	**< 0.001**	7.701	3.743–15.846
No	10 (7.5)	124 (92.5)			
***Wound infection***					
Yes	58 (26.1)	164 (73.9)	0.117	1.768	0.867–3.608
No	11 (16.7)	55 (83.3)			
***Previous foot ulcer***					
Yes	23 (27.1)	62 (72.9)	0.426	1.266	0.709–2.262
No	46 (22.7)	157 (77.3)			
***Amputation***					
Yes	30 (27.8)	78 (72.2)	0.240	1.391	0.802–2.411
No	39 (21.7)	141 (78.3)			

As shown in Table 3, subjects who had proteinuria (*P* < 0.001), bacteraemia (*P* < 0.001), moderate (*P* 0.002) or severe (*P* < 0.001) sonographic vascular stenosis, and low HDL cholesterol (*P* < 0.001) had significantly increased odds of intra-hospital mortality. Mortality was not significantly related to long term glycaemic control as determined by the level of glycated haemoglobin (*P* 0.374).

**Table 3:** Relationship between laboratory and radiologic indices and mortality

	Outcome		*P* value	OR	95% C. I for OR
	Died n (%)	Survived n (%)			
***Proteinuria***					
Yes	39 (36.4)	68 (63.6)	**< 0.001**	2.927	1.672–5.123
No	29 (16.4)	148 (83.6)			
***Glycemic control***					
Good	2 (13.3)	13 (86.7)	0.374	0.502	0.110–2.291
Poor	57 (23.5)	186 (76.5)			
***Blood culture***					
Positive	37 (48.7)	39 (51.3)	**< 0.001**	5.337	2.856–9.972
Negative	24 (15.1)	135 (84.9)			
***Osteomyelitis***					
Yes	20 (26.3)	56 (73.7)	0.350	1.341	0.725–2.483
No	41 (21.0)	154 (79.0)			
***Vascular Stenosis***					
Severe	12 (37.5)	20 (62.5)	**0.002**	4.114	1.657–10.213
Moderate	25 (40.3)	37 (59.7)	**< 0.001**	4.633	2.175–9.870
Mild	15 (21.4)	55 (78.6)	0.125	1.870	0.840–4.163
None	14 (12.7)	96 (87.3)			
***Total cholesterol***					
High	25 (20.5)	97 (79.5)	0.733	0.897	0.480–1.676
Normal	25 (22.3)	87 (77.7)			
***LDL Cholesterol***					
High	29 (22.1)	102 (77.9)	0.746	1.110	0.590–2.089
Normal	21 (20.4)	82 (79.6)			
***HDL Cholesterol***					
Low	54 (34.2)	104 (65.8)	**< 0.001**	6.305	2.728–14.574
Normal	7 (7.6)	85 (92.4)			
***Triglycerides***					
Abnormal	19 (20.2)	75 (79.8)	0.745	0.899	0.473–1.708
Normal	31 (22.0)	110 (78.0)			

Among the co-morbidities that were evaluated, presentation in shock (OR 4.0, *P* < 0.001), cardiac failure (OR 2.8, *P* 0.027), or renal impairment (OR 4.5, *P* < 0.001) were the variables that significantly predicted mortality. Neither hyperglycaemia (*P* 0.127) nor hypoglycaemia (*P* 0.240) significantly predicted mortality (Table 4).

**Table 4:** Relationship between co-morbid complications and mortality

	Outcome		*P* value	OR	95% C. I for OR
	Died n (%)	Discharged n (%)			
***Hypertension***					
Yes	42 (23.6)	136 (76.4)	0.854	0.949	0.545–1.654
No	27 (24.5)	83 (75.5)			
***Shock at presentation***					
Yes	19 (50.0)	19 (50.0)	**< 0.001**	4.000	1.927–8.115
No	50 (20.0)	200 (80.0)			
***Anaemia***					
Yes	44 (28.6)	110 (71.4)	0.051	1.744	0.998–3.046
No	25 (18.7)	109 (81.3)			
***Hyperglycaemic emergency***					
Yes	30 (29.1)	73 (70.9)	0.127	1.538	0.885–2.674
No	39 (21.1)	146 (78.9)			
***Hypoglycaemia***					
Yes	12 (31.6)	26 (68.4)	0.240	1.563	0.742–3.292
No	57 (22.8)	193 (77.2)			
***Stroke***					
Yes	9 (31.0)	20 (69.0)	0.349	1.492	0.646–3.450
No	60 (23.2)	199 (76.8)			
***Cardiac failure***					
Yes	9 (45.0)	11 (55.0)	**0.027**	2.836	1.123–7.164
No	60 (22.4)	208 (77.6)			
***Renal impairment***					
Yes	29 (49.2)	30 (50.8)	**< 0.001**	4.543	2.459–8.395
No	40 (17.5)	188 (82.5)			

On multivariate regression, the presence of bacteraemia and renal impairment were independent predictors of mortality in the study population (OR 5.1, *P* < 0.001 and OR 2.8, *P* < 0.006 respectively) (Table 5).

**Table 5:** Multivariate predictors of mortality

Variable		B	*P* value	OR	95% C. I for OR	
					Lower	Upper
	Age ≥ 45	1.018	0.145	2.768	0.703	10.903
	Ulcer > 1 month duration	0.555	0.168	1.742	0.791	3.835
	Peripheral artery disease	0.315	0.383	1.370	0.675	2.778
	Positive blood culture	1.620	**< 0.001**	5.053	2.572	9.928
	Anaemia	0.020	0.954	1.020	0.513	2.031
	Cardiac failure	0.771	0.202	2.161	0.661	7.067
	Renal impairment	1.043	**0.006**	2.838	1.349	5.971

## Discussion

Diabetic foot ulcers are responsible for considerable morbidity and mortality of diabetic patients. Both hospitalized and patients attending ambulatory care settings who have DFUs are shown in worldwide studies to have higher mortality rates than patients without [19, 20]. Identification of factors that contribute to this high mortality – the focus of this article - may go a long way in improving survival in patients with DFU.

Intra-hospital mortality of 21.4% was recorded in this observational study. This finding is higher than what was reported previously, both within and outside Nigeria. For instance, Edo et al. [21] reported a mortality rate of 14% in Benin (Southern Nigeria). Similarly, Rigato et al. in a meta-analysis of studies conducted in many parts of Africa reported a mortality of 14% [9], an Indonesian study observed mortality rate of 10.7% [22], while much lower mortality rates of 4 and 2% were respectively reported in Manchester, England [23] and the United States (US) [24]. On the other hand, Ekpebegh et al. [7] in 2009 reported a much higher in-hospital mortality of 40.5% in a single-center study conducted in Lagos, South-western Nigeria. These observations suggest that although there are wide geographical variations in DFU-related mortalities, they appear to be worrisomely higher in Nigeria than in most parts of the world. Several reasons may account for the disproportionately high mortality observed in this study. First, the majority of the patients (72%) enrolled in this study did not access routine diabetes care at the study centres but were referred from primary and secondary health facilities which are usually lacking in requisite expertise and facilities. Secondly, delay in hospital presentation is another plausible explanation for this observed mortality. For instance, we observed that subjects with ulcer duration longer than 1 month had more than twice the odds of dying than those with a shorter duration of ulcer. It is a fact that the longer an ulcer lasts, the higher the likelihood of wound infection that may progress to sepsis. This notion is supported by the fact that sepsis, as evidenced by positive blood culture, remained as an independent predictor of mortality after adjusting for other confounding variables in the study by Nanna Panday et al. [25] The much lower mortality in the fore mentioned studies from Manchester and the US may reflect the impact of an advanced healthcare system available in these countries on disease outcomes.

In this study, we found that age was significantly associated with mortality, with the highest mortality recorded among individuals 65 years and above. Foryoung et al. in Cameroon, Boyco et al. in the US, Katz et al. in Israel and Lynar et al. in Australia reported similar findings [5, 11, 26, 27]. This finding may be explained by the fact that the prevalence of medical comorbidities that may lead to organ dysfunctions and death tend to increase with advancing age. Furthermore, micro and macrovascular diabetes complications are usually more life-threatening when they present in older than younger persons. It has been previously observed that diabetes in older adults is linked to higher mortality, reduced functional status, and increased risk of institutionalization [28]. On the contrary, some researchers did not observe any association between age and DFU-related mortality [23]. The observed relationship between age and mortality however disappeared on multivariate analysis, suggesting that the effect of advancing age on DFU-related mortality is indirect, probably through other diabetes complications. This view is supported by the fact that the presence of some co-morbid complications such as shock, cardiac failure, and renal impairment was significantly associated with increased mortality in our study. It is also noteworthy that the mean age of those hospitalised for DFU in this study (57.2 ± 11.9 yrs.) belongs to the working-age population, in contrast to what obtains in developed nations where the elderly (70 years and above) predominate [4]. The public health implication is that this may contribute to the depletion of the Nigerian labour force with the resultant negative effect on national output and national income.

We observed a proportional increase in mortality with increasing ulcer grade in this study. For instance, subjects with advanced Wagner grade ulcers (≥ grade 3) were almost eight times more likely to die compared to those with lower grade ulcers. This is not surprising since higher Wagner foot ulcer grade indicates more advanced disease and increased probability of sepsis which could predispose the patient to multi-organ dysfunction. An earlier Nigerian study also observed increased mortality in patients who presented with Wagner grade 4 and 5 ulcers [7]. In contrast, Jeraman et al. [19] did not find an association between Wagner grade and mortality probably because a higher percentage of the study subjects presented with Wagner grade 1 and 2 ulcers. It is also noteworthy, that the observed association between Wagner grade and mortality in our study was not independent of other variables as ulcer grade failed to emerge as a significant predictor of mortality when controlled for other potential predictors in multivariate analysis. The low frequency of Wagner grade 1 ulcer and corresponding higher mortality of this subset than Wagner grade 2 recorded in this study may be a result of the drawback of the Wagner grading system’s inability to address ischemia and infection which were contributors to mortality in this study [29].

Gender was not observed to significantly influence mortality, neither was cigarette smoking status. However, a longer duration of DM was a significant predictor of mortality. A similar finding was also reported by Martins Mendez et al. [30] Long duration of DM is associated with the development of micro and macrovascular complications and death [31].

We observed that the presence of peripheral arterial disease, as well as foot gangrene, were significant predictors of death. Subjects with gangrene had eight times higher probability of death than those without. There was also a significant association between the severity of PAD as detected on vascular imaging and mortality, such that subjects with moderate or severe stenosis had significantly higher odds of death compared to those with mild or no stenosis. Peripheral arterial disease in DFUs has been reported to be associated with severe adverse outcomes, which include the lower probability of healing, prolonged healing times, increased incidence of ulcer recurrence, amputations, and higher mortality [32, 33]. Presence of PAD may also be a pointer to atherosclerosis in other vessels including coronary vessels, which place such patients at higher risk of myocardial infarction and sudden cardiac deaths.

Laboratory indices that were predictive of mortality in the cohort of patients in this study include proteinuria, positive blood cultures, and low HDL cholesterol. In a meta-analysis of randomized clinical trials of type 2 DM, selection for renal disease which was defined by either decline in renal function or presence of proteinuria signalled important mortality risk [34]. Furthermore, Aragon-Sanchez et al. reported albuminuria, anaemia, and leukocytosis as predictors of in-hospital mortality in patients admitted for DFU [35].

Positive blood culture is indicative of sepsis and the attendant systemic inflammatory response which carries a high risk of thromboses and organ dysfunction which have been associated with higher mortality in sufferers both generally and specifically in those with DFUs [36, 37].

Although diabetic patients with DFU have been observed to have a higher prevalence of cardiovascular risk factors such as hypercholesterolemia, hypertriglyceridemia, hyperuricemia, and proteinuria [38], our observation of an association between mortality in hospitalized patients with DFU and low HDL cholesterol appears to be novel. Diabetes mellitus is associated with low HDL which is an established risk factor for cardiovascular disease in diabetic patients [39]. Furthermore, cardiovascular disease is reported to be the most prevalent cause of death in diabetic patients [40, 41].

Co-morbid conditions that predicted mortality in this study were cardiac failure, shock, and renal impairment. Diabetic patients have a greater than a two-fold risk of developing cardiac failure and the prognosis is worse in diabetic patients [42], which may explain the association between cardiac failure and mortality in this study. The impact of shock on mortality is well documented. It inhibits the perfusion of vital organs. Sang WookYi [43] in a Korean study reported an association between low systolic blood pressure and vascular mortality. Similarly, Tringali described an association between reduction in diastolic blood pressure below 70 mm of mercury and all-cause mortality [44]. Renal impairment persisted as an independent predictor of mortality on multivariate analysis. The association between mortality and renal impairment may be multifactorial, namely worsening nephrotoxicity from antibiotherapy as well as the underlying cause which in the setting of hospitalization for DFU may be sepsis. Furthermore, renal impairment may contribute directly to mortality in the diabetic patient by promoting cardiovascular risk factors such as hypertension, insulin resistance, oxidative stress, endothelial dysfunction, and inflammation.

A recent study from Saudi Arabia reported similar findings of nephropathy being an independent risk factor for all-cause mortality among patients with diabetic foot complications [6].Given the high mortality of hospitalized DFU patients in our setting, identification of the above-stated prognosticators is crucial to guide strategies for improved outcomes.

## Conclusions

This study demonstrated that intra-hospital mortality from DFU in Nigeria is high and is related to many local and systemic variables. Ulcer-related determinants of mortality were longer duration of ulcer before hospitalization, higher Wagner grades (≥ 3) and foot gangrene; while systemic factors that significantly predicted mortality include older age, longer diabetes duration, peripheral artery disease, shock, bacteraemia, renal impairment, low HDL- cholesterol, and cardiac failure. Renal impairment and bacteraemia were independent determinants of mortality.

Future studies need to explore the benefits of prompt attention to some of these identified factors to reduce DFU-related mortality in Nigeria. In the interim, patient education on the importance of early presentation of foot lesions to the hospital needs to be pursued. There is also a need to focus more attention on primary and secondary level hospitals as our study suggests that most patients hospitalized for DFU across Nigerian tertiary hospitals access diabetes care at these lower cadres of healthcare delivery. Training of the health care practitioners at this level on risk factor identification for foot ulcers, prompt management of foot lesions, and timely referral to multi-disciplinary specialist care centres might go a long way in stemming the current tide.

### Limitations of this study

The mortality rate observed in this study may not represent the actual figures since the outcome in about 10% who discharged against medical advice could not be ascertained. However, since nearly half of those patients opted out of care due to refusal of amputation, indicating that they had advanced ulcers and therefore increased risk of mortality, our finding might have underestimated the actual reality. Secondly, the impact that variations in expertise and facilities in the participating study centres had on our findings cannot be determined. Thirdly, the possibility that other co-morbidities that were not evaluated for influenced the findings in this study cannot be ruled out. Lastly, like in all cross-sectional studies, it is impossible to establish a cause and effect relationship in this study.

## Data Availability

Data from this study can be obtained from the corresponding author.

## Abbreviations

DFU: Diabetic foot ulcers
LEA: Lower extremity amputation
IWDGF: International Working Group on Diabetic Foot
PAD: Peripheral artery disease
DM: Diabetes mellitus
HDL: High-density lipoprotein cholesterol
LDL: Low-density lipoprotein cholesterol
TG: Triglycerides

## References

[1] Federation ID (2017). International diabetes atlas.

[2] Morbach S, Furchert H, Gröblinghoff U, Hoffmeier H, Kersten K, Klauke G-T (2012). Long-term prognosis of diabetic foot patients and their limbs: amputation and death over the course of a decade. Diabetes Care.

[3] Ghanassia E, Villon L, Thuan Dit Dieudonné J-F, Boegner C, Avignon A, Sultan A (2008). Long-term outcome and disability of diabetic patients hospitalized for diabetic foot ulcers: a 6.5-year follow-up study. Diabetes Care.

[4] Chammas NK, Hill RLR, Edmonds ME (2016). Increased mortality in diabetic foot ulcer patients: the significance of ulcer type. J Diabetes Res.

[5] Boyko EJ, Ahroni JH, Smith DG, Davignon D (1996). Increased mortality associated with diabetic foot ulcer. Diabet Med.

[6] Al-Rubeaan K, Almashouq MK, Youssef AM, Al-Qumaidi H, Al Derwish M, Ouizi S (2017). All-cause mortality among diabetic foot patients and related risk factors in Saudi Arabia. PLoS One.

[7] Ekpebegh CO, Iwuala SO, Fasanmade OA, Ogbera AO, Igumbor E, Ohwovoriole AE (2009). Diabetes foot ulceration in a Nigerian hospital: in-hospital mortality in relation to the presenting demographic, clinical and laboratory features. Int Wound J.

[8] Ogbera AO, Chinenye S, Onyekwere A, Fasanmade O (2007). Prognostic indices of diabetes mortality. Ethn Dis.

[9] Rigato M, Pizzol D, Tiago A, Putoto G, Avogaro A, Fadini GP (2018). Characteristics, prevalence, and outcomes of diabetic foot ulcers in Africa. A systemic review and meta-analysis. Diabetes Res Clin Pract.

[10] Xie Y, Zhang H, Ye T, Ge S, Zhuo R, Zhu H (2017). The geriatric nutritional risk index independently predicts mortality in diabetic foot ulcers patients undergoing amputations. J Diabetes Res.

[11] Katz DE, Friedman ND, Ostrovski E, Ravid D, Amrami N, Avivi D (2016). Diabetic foot infection in hospitalized adults. J Infect Chemother.

[12] Ricci L, Scatena A, Tacconi D, Ventoruzzo G, Liistro F, Bolognese L (2017). All-cause and cardiovascular mortality in a consecutive series of patients with diabetic foot osteomyelitis. Diabetes Res Clin Pract.

[13] Essackjee Z, Gooday C, Nunney I, Dhatariya K (2017). Indicators of prognosis for admissions from a specialist diabetic foot clinic: a retrospective service improvement exercise. J Wound Care.

[14] Brennan MB, Hess TM, Bartle B, Cooper JM, Kang J, Huang ES (2017). Diabetic foot ulcer severity predicts mortality among veterans with type 2 diabetes. J Diabetes Complicat.

[15] Ugwu E, Adeleye O, Gezawa I, Okpe I, Enamino M, Ezeani I (2019). Burden of diabetic foot ulcer in Nigeria: Current evidence from the multicenter evaluation of diabetic foot ulcer in Nigeria. World J Diabetes.

[16] Lipsky BA, Aragon-Sanchez J, Diggle M, Embil J, Kono S, Lavery L (2016). IWGDF guidance on the diagnosis and management of foot infections in persons with diabetes. Diabetes Metab Res Rev.

[17] Wagner FW (1981). The dysvascular foot: a system for diagnosis and treatment. Foot Ankle.

[18] Al-Qaisi M, Nott DM, King DH, Kaddoura S (2009). Ankle brachial pressure index (ABPI): an update for practitioners. Vasc Health Risk Manag.

[19] Jeyaraman K, Berhane T, Hamilton M, Chandra AP, Falhammar H (2019). Mortality in patients with diabetic foot ulcer: a retrospective study of 513 cases from a single Centre in the Northern Territory of Australia. BMC Endocr Disord.

[20] Moulik PK, Mtonga R, Gill GV (2003). Amputation and mortality in new-onset diabetic foot ulcers stratified by etiology. Diabetes Care.

[21] Edo A, Edo G, Ezeani I (2013). Risk factors, ulcer grade and management outcome of diabetic foot ulcers in a Tropical Tertiary Care Hospital. Niger Med J.

[22] Pemayun TGD, Naibaho RM (2017). Clinical profile and outcome of diabetic foot ulcer, a view from tertiary care hospital in Semarang, Indonesia. Diabet Foot Ankle.

[23] Oyibo SO, Jude EB, Tarawneh I, Nguyen HC, Armstrong DG, Harkless LB (2001). The effects of ulcer size and site, patient’s age, sex and type and duration of diabetes on the outcome of diabetic foot ulcers. Diabet Med.

[24] Skrepnek GH, Mills JL, Armstrong DG (2015). A diabetic emergency one million feet long: disparities and burdens of illness among diabetic foot ulcer cases within emergency departments in the United States, 2006–2010. PLoS One.

[25] Nannan Panday RS, Lammers EMJ, Alam N, Nanayakkara PWB (2019). An overview of positive cultures and clinical outcomes in septic patients: a sub-analysis of the Prehospital Antibiotics Against Sepsis (PHANTASi) trial. Crit Care.

[26] Foryoung JB, Ditah C, Nde Fon P, Mboue-Djieka Y, Nebongo DN, Mbango ND (2018). Long-term mortality in outpatients with type 2 diabetes in a reference hospital in Cameroon: a retrospective cohort study. BMJ Open.

[27] Lynar SA, Robinson CH, Boutlis CS, Commons RJ. Risk factors for mortality in patients with diabetic foot infections: a prospective cohort study. Intern Med J. 2019;49(7):867-73. 10.1111/imj.14184 30515957

[28] Brown AF, Mangione CM, Saliba D, Sarkisian CA (2003). Guidelines for improving the care of the older person with diabetes mellitus. J Am Geriatr Soc.

[29] Frykberg RG (2002). Diabetic foot ulcers: pathogenesis and management. Am Fam Physician.

[30] Martins-Mendes D, Monteiro-Soares M, Boyko EJ, Ribeiro M, Barata P, Lima J (2014). The independent contribution of diabetic foot ulcer on lower extremity amputation and mortality risk. J Diabetes Complicat.

[31] Zoungas S, Woodward M, Li Q, Cooper ME, Hamet P, Harrap S (2014). Impact of age, age at diagnosis and duration of diabetes on the risk of macrovascular and microvascular complications and death in type 2 diabetes. Diabetologia..

[32] Armstrong DG, Cohen K, Courric S, Bharara M, Marston W (2011). Diabetic foot ulcers and vascular insufficiency: our population has changed, but our methods have not. J Diabetes Sci Technol.

[33] Costa RHR, Cardoso NA, Procopio RJ, Navarro TP, Dardik A, de Loiola Cisneros L (2017). Diabetic foot ulcer carries high amputation and mortality rates, particularly in the presence of advanced age, peripheral artery disease and anemia. Diabetes Metab Syndr.

[34] Barkoudah E, Skali H, Uno H, Solomon SD, Pfeffer MA (2012). Mortality rates in trials of subjects with type 2 diabetes. J Am Heart Assoc.

[35] Aragon-Sanchez J, Lazaro-Martinez JL, Garcia-Alvarez Y, Morales EG, Hernandez-Herrero MJ (2014). Albuminuria is a predictive factor of in-hospital mortality in patients with diabetes admitted for foot disease. Diabetes Res Clin Pract.

[36] Jacobi J (2002). Pathophysiology of sepsis. Am J Health Syst Pharm.

[37] Harris CM, Abougergi MS, Wright S (2019). Clinical outcomes among morbidly obese patients hospitalized with diabetic foot complications. Clin Obes.

[38] Pinto A, Tuttolomondo A, Di Raimondo D, Fernandez P, La Placa S, Di Gati M (2008). Cardiovascular risk profile and morbidity in subjects affected by type 2 diabetes mellitus with and without diabetic foot. Metab Clin Exp.

[39] Sharif S, van der Graaf Y, Nathoe HM, de Valk HW, Visseren FL, Westerink J (2016). HDL cholesterol as a residual risk factor for vascular events and all-cause mortality in patients with type 2 diabetes. Diabetes Care.

[40] Leon BM, Maddox TM (2015). Diabetes and cardiovascular disease: epidemiology, biological mechanisms, treatment recommendations and future research. World J Diabetes.

[41] Einarson TR, Acs A, Ludwig C, Panton UH (2018). Prevalence of cardiovascular disease in type 2 diabetes: a systematic literature review of scientific evidence from across the world in 2007–2017. Cardiovasc Diabetol.

[42] Nichols GA, Hillier TA, Erbey JR, Brown JB (2001). Congestive heart failure in type 2 diabetes: prevalence, incidence, and risk factors. Diabetes Care.

[43] Yi S-W, Hong S, Ohrr H (2015). Low systolic blood pressure and mortality from all-cause and vascular diseases among the rural elderly in Korea; Kangwha cohort study. Medicine (Baltimore).

[44] Tringali S, Oberer CW, Huang J (2013). Low diastolic blood pressure as a risk for all-cause mortality in VA patients. Int J Hypertens.

